# Comprehensive analysis of prognostic factors and clinical implications of pneumonectomy for destroyed lung

**DOI:** 10.3389/fsurg.2026.1907176

**Published:** 2026-08-26

**Authors:** Shanbo Zheng, Akbar Anwar, Wancheng Zhong, Xiaogang Yan

**Affiliations:** 1 Department of Thoracic Surgery, Xinjiang Uygur Autonomous Region Sixth People’s Hospital, Urumqi, Xinjiang Uygur Autonomous Region, China; 2 Department of Thoracic Surgery, Fudan University Shanghai Cancer Center, Shanghai, China

**Keywords:** destroyed lung, pneumonectomy, postoperative complication, prognostic factor, pulmonary infectious diseases

## Abstract

**Background:**

Evaluate the efficacy and safety of pneumonectomy in the treatment of destroyed lung caused by benign diseases, and identify the risk factors for postoperative complications.

**Methods:**

We retrospectively analyzed 158 patients with destroyed lung caused by benign diseases who underwent pneumonectomy in a single center from January 2014 to December 2024. Cases were assigned to tuberculosis (TB) group and non-tuberculosis (non-TB) group. Clinical characteristics and postoperative complications were analyzed and compared between groups. Univariate and multivariate analyses were performed to investigate the risk factors of pneumonectomy.

**Results:**

A total of 125 TB and 33 non-TB patients were included. All patients with preoperative hemoptysis recovered after surgery. Major postoperative complications were observed in 32 patients (20.3%), and perioperative deaths were noted in three patients (1.9%), who were from the TB group. The TB group had a longer duration of surgery and length of stay in the intensive care unit (ICU), a higher proportion of diabetes and postoperative complications (24.0% vs. 6.1%, *p* = 0.027) than the non-TB group. Multivariate logistic analyses indicated diabetes [odds ratio [OR]: 4.680; 95% conﬁdence interval [CI]: 1.578–13.881; *p* = 0.005] and duration of surgery (OR: 1.005; 95% CI: 1.001–1.010; *p* = 0.036) were the independent risk factors of postoperative complications of pneumonectomy.

**Conclusion:**

Pneumonectomy is effective for destroyed lung caused by benign diseases. Patients with TB destroyed lung have a higher risk of postoperative complications. Controlling perioperative blood glucose and reducing duration of surgery may reduce the risk of perioperative complications.

## Introduction

Destroyed lung refers to an end-stage lesion induced by various chronic inﬂammatory pulmonary diseases, which can lead to loss of pulmonary function, recurrent infections, and even life-threatening massive hemoptysis (1, 2). Pneumonectomy is a crucial treatment for destroyed lung; however, it is a major surgical procedure with a high risk of postoperative complications (3–5). The etiologies of destroyed lung include tuberculosis (TB) and non-tuberculous (non-TB) diseases, such as bronchiectasis and bronchocele (6–8). These two etiological categories differ substantially in their pathophysiological mechanisms. Chronic TB infection is characterized by granulomatous inflammation, caseous necrosis, fibrosis, and calcification, which often result in dense pleural adhesions, hilar fibrosis, and bronchial distortion. These changes can make surgical dissection extremely challenging and increase the risk of intraoperative bleeding and postoperative complications. In contrast, non-TB etiologies such as bronchiectasis and bronchocele typically involve less extensive fibrosis and may be associated with fewer technical difficulties during surgery. These pathophysiological differences may account for the distinct surgical outcomes observed between the two groups (9, 10). However, comparative studies on pneumonectomy for these two categories of diseases remain scarce.

Therefore, we retrospectively analyzed cases of pneumonectomy performed for destroyed lung caused by benign diseases. The objectives were to explore the differences in clinical characteristics of pneumonectomy between TB and non-TB destroyed lung, and to identify the risk factors for complications, thereby improving perioperative management.

## Methods

### Study design and participants

We retrospectively analyzed 158 patients with destroyed lungs caused by benign diseases who underwent pneumonectomy from January 2014 to December 2024 in Xinjiang Uygur Autonomous Region Sixth People's Hospital, a specialized center for tuberculosis and other infectious diseases. In this study, destroyed lung was defined as pulmonary destruction caused by various benign diseases, with chest imaging findings showing extensive destruction of lung tissue and massive proliferation of fibrous tissue, as described in previous studies. Indications for pneumonectomy included: (1) unilateral destroyed lung, absence of lung lesions or only small lesions in the contralateral lung; (2) massive hemoptysis with poor response to medical or interventional treatment; (3) history of destroyed lung related recurrent infections; (4) antituberculosis treatment was ineffective or intolerable; (5) adequate cardiopulmonary function to tolerate surgery. Patients were enrolled consecutively, and those with concurrent lung tumors were excluded. Patients included in the analysis were assigned two groups according to whether pulmonary tuberculosis is diagnosed: tuberculosis (TB) group and non-tuberculosis (non-TB) group. The study design complies with the Helsinki statement on research ethics. The Institutional Review Board of Xinjiang Uygur Autonomous Region Sixth People's Hospital approved the retrospective study. The requirement for informed consent was waived because only anonymized data were included in the present study.

### Clinical parameters and follow-up

Clinical data collected from patients at baseline included sex, age, present illness history, past medical history, smoking history, comorbidities, body mass index (BMI), routine laboratory examination, pulmonary function test, chest computed tomography, secondary or primary multidrug-resistant TB, pathological diagnosis, and surgical details. Multidrug-resistant TB (MDR-TB) was defined as TB caused by a Mycobacterium tuberculosis strain that was resistant to rifampin and isoniazid; extensively drug-resistant TB (XDR-TB) was defined as MDR-TB with additional resistance to both any fluoroquinolone and any second-line injectable drug (11). Pneumonectomy was performed via conventional surgical approaches, which included either thoracotomy or thoracoscopy, with the specific approach determined by the operating surgeon. Open thoracotomy was performed through a muscle-sparing anterolateral incision (approximately 10–15 cm in length). Postoperative complications were defined as those occurring during hospitalization or within 30 days postoperatively and were classified according to the Clavien-Dindo system. Grade I complications (deviation from normal recovery without need for pharmacological or procedural intervention) were classified as mild complications. Grade ≥ II complications, which required pharmacological treatment (Grade II), surgical/endoscopic/radiological intervention (Grade III), or were life-threatening requiring ICU management (Grade IV) or resulted in death (Grade V), were classified as moderate-to-severe complications and constituted the primary outcome endpoint for analysis. Follow-up was completed in all patients by telephone or outpatient clinic using a structured questionnaire to ensure standardized data collection.

### Statistical analyses

Numbers and percentages were provided for categorical variables, whereas mean ± standard deviation (SD) or median was provided for quantitative variables. Difference in proportions was analyzed by Pearson's chi-squared test or Fisher's exact test. The normality of continuous variables was assessed using the Shapiro–Wilk test. The Mann–Whitney U test was utilized to analyze continuous variables between the two groups when the data were not normally distributed, while Student's t-test was used for normally distributed variables. Binary logistic regression analysis was used to identify predictors of postoperative complications. Variables with a univariate *p*-value < 0.10 were considered candidates for multivariable analysis, along with clinically relevant variables (e.g., age, diabetes, and pulmonary function) identified from previous literature. A forward stepwise selection method (entry criterion: *p* < 0.05; removal criterion: *p* > 0.10) was used to identify independent risk factors for postoperative complications. *P* values < 0.05 were considered statistically significant. Data were analyzed using the SPSS software (SPSS version 22, SPSS Inc., Chicago, IL, USA).

## Results

### Clinical characteristics of patients

A total of 158 patients were enrolled in this study. Among them, 125 (79.1%) were diagnosed with tuberculosis via preoperative bacteriological examination or postoperative pathological examination and categorized into the tuberculosis (TB) group. The remaining 33 (20.9%) patients were assigned to the non-tuberculosis (non-TB) group. Table 1 shows the etiology of destroyed lung in detail.

**Table 1:** Etiology of destroyed lung.

Etiology	Number	Percentage
Tuberculosis	**125**	**79.1%**
Tuberculosis	108	68.4%
Tuberculosis + Aspergillosis	16	10.1%
Tuberculosis + Bronchiectasis	1	0.6%
non-Tuberculosis	**33**	**20.9%**
Bronchocele	12	7.6%
Bronchiectasis	11	7.0%
Congenital pulmonary dysplasia	4	2.5%
Congenital pulmonary dysplasia + Bronchiectasis	2	1.3%
Chronic septic pulmonary abscess	2	1.3%
Pulmonary aspergillosis	1	0.6%
Organizing Pneumonia	1	0.6%

The bold value shows the etiologies of destroyed lung (tuberculous vs. non-tuberculous) with case numbers and percentages for each item.

There were 77 males and 81 females with a mean age of 37.09 ± 13.64 years (Table 2). Of the patients, 120 underwent left-sided surgery and 38 right-sided surgery. Only 3 patients underwent video-assisted thoracoscopic surgery (VATS); however, due to the small number, a comparative analysis between the two approaches was not performed. Forty-one patients had preoperative hemoptysis, which resolved in all cases after surgery. There were 16 patients with diabetes mellitus. Compared with the non-TB group, the TB group exhibited a significantly higher prevalence of diabetes mellitus, whereas no statistically significant differences were noted in other clinical characteristics. Additionally, the TB group had a longer mean operation time and an extended length of ICU stay.

**Table 2:** Demographic and clinical baseline characteristics of patients with Tuberculosis vs. non-Tuberculosis destroyed lung pneumonectomy.

Characteristics	Total (*n* = 158)	Tuberculosis (*n* = 125)	non-Tuberculosis (*n* = 33)	*P* value
Age (year)	37.09 ± 13.64	37.22 ± 14.26	36.58 ± 11.20	0.809
Sex				0.672
Male %	77 (48,7)	62 (49.6)	15 (45.5)	
Female %	81 (51.3)	63 (50.4)	18 (54.5)	
Operation side				0.977
Left %	120 (75.9)	95 (76.0)	25 (75.8)	
Right %	38 (24.1)	30 (24.0)	8 (24.2)	
Emergency admission %	37 (23.4)	27 (21.6)	10 (30.3)	0.294
Massive hemoptysis %	41 (25.9)	33 (26.4)	8 (24.2)	0.901
BMI (kg/m^2^)	20.69 ± 3.71	20.40 ± 3.67	21.80 ± 3.73	0.058
Smoking history %	61 (38.6)	48 (38.4)	13 (39.4)	0.917
Comorbidities %	29 (18.4)	25 (20.0)	4 (12.1)	0.448
Diabetes mellitus %	16 (10.1)	16 (12.8)	0 (0)	0.030
Other disease %	16 (10.1)	12 (9.6)	4 (12.1)	0.746
MDR/XDR %		28 (22.4)		
Aspergillus positive %	23 (14.6)			
TB sputum positive %		13 (10.4)		
PFT				
FVC % Pred	56.27 ± 9.96	55.31 ± 10.15	63.95 ± 2.76	0.259
FEV1% pred	49.35 ± 13.17	50.35 ± 13.60	40.85 ± 0.64	0.349
MVV % pred	48.64 ± 20.21	49.34 ± 21.09	42.00 ± 8.34	0.638
HB (g/L)	118.45 ± 20.21	118.86 ± 20.51	116.54 ± 19.08	0.612
ALB (g/L)	38.94 ± 4.77	38.78 ± 4.70	39.63 ± 5.11	0.425
CRP (mg/L)	30.89 ± 45.72	32.02 ± 47.26	25.09 ± 37.61	0.581
ESR (mm/h)	49.94 ± 36.00	50.95 ± 36.23	44.67 ± 35.28	0.500
Operation factors				
Duration of surgery (minute)	202.69 ± 81.15	210.44 ± 79.75	173.33 ± 80.88	0.019
Mean bleeding volume (mL)	742.78 ± 1,036.72	742.80 ± 852.76	742.73 ± 1,566.91	>0.999
Type of suture				>0.999
Staple %	152 (96.2)	120 (96.0)	32 (97.0)	
Suture %	6 (3.8)	5 (4.0)	1 (3.0)	
Postoperative hospital stay (day)	19.41 ± 7.49	19.68 ± 7.40	18.36 ± 7.88	0.371
ICU stay (day)	0.93 ± 2.16	1.11 ± 2.34	0.24 ± 1.06	0.002
Length of stay (day)	35.25 ± 11.69	34.70 ± 11.67	37.33 ± 11.70	0.250

BMI, body mass index; MDR/XDR, multidrug resistant tuberculosis/extensively drug-resistant; TB, Tuberculosis; PFT, pulmonary function test; FVC % pred, forced vital capacity of predicted value; FEV1% pred, forced expiratory volume in one second of predicted value; MVV % pred, maximum voluntary ventilation of predicted value; HB, hemoglobin; ALB, albumin; CRP, C-reaction protein; ESR, erythrocyte sedimentation rate; ICU, Intensive Care Unit.

### Postoperative complications

Overall, 32 patients (20.3%) experienced moderate-to-severe postoperative complications (Clavien-Dindo Grade ≥ II). Grade I (mild) complications were not included in the primary outcome analysis. The most frequently observed complication was pulmonary infection and arrhythmia (*n* = 9, 5.70%), followed by empyema (*n* = 6, 3.80%), bronchopleural fistula (*n* = 3, 1.90%), postoperative bleeding requiring reoperation (*n* = 2, 1.27%) and others. (Table 3) Patients with pulmonary infection were administered anti-infective therapy and ventilator support. All patients achieved clinical recovery, with the exception of one case who ultimately succumbed to the disease. All patients with empyema achieved clinical recovery following closed thoracic drainage and thoracic irrigation with normal saline (or distilled water in selected cases based on culture and sensitivity results). Among the patients with bronchopleural fistula, two cases had small fistulas that gradually healed after closed thoracic drainage. In contrast, one case with a large fistula underwent thoracoplasty one month after pneumonectomy and achieved satisfactory recovery. Three perioperative deaths occurred within 30 days after surgery. The causes of death were postoperative hemorrhage, severe pulmonary infection, and contralateral spontaneous pneumothorax, respectively.

**Table 3:** Outcomes of patients with Tuberculosis vs. non-Tuberculosis destroyed lung pneumonectomy.

Postoperative complication	Total (*n* = 158)	Tuberculosis (*n* = 125)	non-Tuberculosis (*n* = 33)	*P* value	95% confidence interval
Total	32	30	2	0.027	1.201–21.66
Pulmonary infection	9	9	0	0.206	0.6846-∞
Arrhythmia	9	8	1	0.686	0.3080–24.99
Empyema	6	5	1	>0.999	0.1703–16.16
Bronchopleural fistula	3	3	0	>0.999	0.2276-∞
Abnormal liver function	3	3	0	>0.999	0.2276-∞
Postoperative bleeding requiring reoperation	2	2	0	>0.999	0.1215-∞
Surgical site infection	1	1	0	>0.999	0.02933-∞
Spontaneous pneumothorax	1	1	0	>0.999	0.02933-∞
Death (within 30 days)	3	3	0	>0.999	0.2276-∞
Death (within 90 days)	7	7	0	0.346	0.4705-∞

The incidence rate of major postoperative complications in the TB group was significantly higher than that in the non-TB group (23.2% vs. 6.1%, *P* = 0.027). Nevertheless, the incidence rates of specific complications were not significantly different between the TB and non-TB group. There was also no statistically significant difference in the postoperative mortality rate within 30 days between the two groups.

### Risk factor of postoperative complications

Univariate analysis showed that postoperative complications were associated with diabetes mellitus, pulmonary tuberculosis, and duration of surgery (Table 4). Multivariate Logistic regression analysis indicated that diabetes mellitus [odds ratio [OR]: 4.680; 95% conﬁdence interval [CI]: 1.578–13.881; *p* = 0.005] and duration of surgery (OR: 1.005; 95% CI: 1.001–1.010; *p* = 0.036) were independent risk factors for postoperative complications (Table 5). Although tuberculosis was associated with postoperative complications in univariate analysis (*p* = 0.023), it did not remain an independent predictor in the multivariable model after adjustment for diabetes and duration of surgery. This suggests that the higher complication rate observed in the TB group is largely mediated by the higher prevalence of diabetes and the greater surgical complexity (reflected by longer operative duration) in these patients.

**Table 4:** Univariable analysis for factors associated of postoperative complications.

Variables	With complications (*n* = 32)	Without complications (*n* = 126)	*P* value
Age (year)	41.19 ± 14.40	36.05 ± 13.30	0.057
Sex			0.177
Male %	19 (59.4)	58 (46.0)	
Female %	13 (40.6)	68 (54.0)	
Operation side			0.888
Left %	24 (75.0)	96 (76.2)	
Right %	8 (25.0)	30 (23.8)	
Emergency admission %	7 (21.9)	30 (23.8)	0.817
Massive hemoptysis %	11 (34.4)	30 (23.8)	0.223
BMI (kg/m^2^)	20.67 ± 4.23	20.70 ± 3.60	0.97
Smoking history %	15 (46.9)	46 (36.5)	0.282
Comorbidities %	9 (28.1)	19 (15.1)	0.118
Diabetes mellitus %	8 (25.0)	8 (6.3)	0.005
Tuberculosis %	29 (90.6)	96 (76.2)	0.023
Aspergillus positive %	6 (18.8)	17 (13.5)	0.416
Duration of surgery (minute)	231.88 ± 95.63	195.28 ± 75.70	0.022
Mean bleeding volume (mL)	1,142.50 ± 1,657.88	641.27 ± 785.49	0.105
Type of suture			>0.999
Staple %	31 (96.9)	121 (96.0)	
Suture %	1(3.1)	5(4.0)	

BMI, body mass index. Variables included in the multivariable model were, age, sex, diabetes mellitus, tuberculosis, and duration of surgery (variables with *p* < 0.10 in univariate analysis).

**Table 5:** Multivariable logistic regression analysis for key factors associated of postoperative complications.

Variables	*P* value	Adjusted OR (95% CI)
Diabetes mellitus	0.005	4.680 (1.578–13.881)
Duration of surgery	0.036	1.005 (1.001–1.010)

OR, odds ratio; CI, conﬁdence interval.

Given the large number and high incidence of complications in the TB group, we further analyzed the risk factors for complications in this group. Univariate analysis showed that postoperative complications were associated with age and diabetes mellitus (Table 6). Multivariate Logistic regression analysis indicated that diabetes mellitus [odds ratio [OR]: 3.955; 95% conﬁdence interval [CI]: 1.335–11.714; *p* = 0.013] was the independent risk factor for postoperative complications (Table 7).

**Table 6:** Univariable analysis for factors associated of postoperative complications in Tuberculosis.

Variables	With complications (*n* = 30)	Without complications (*n* = 95)	*P* value
Age (year)	41.70 ± 14.71	35.81 ± 13.89	0.048
Sex			0.375
Male %	17 (56.7)	45 (47.4)	
Female %	13 (43.3)	50 (52.6)	
Operation side			0.922
Left %	23 (76.7)	72 (75.8)	
Right %	7 (23.3)	23 (24.2)	
Emergency admission %	7 (23.3)	20 (21.1)	0.791
Massive hemoptysis %	10 (33.3)	23 (24.2)	0.323
BMI (kg/m^2^)	20.68 ± 4.31	20.33 ± 3.48	0.661
Smoking history %	14 (46.7)	34 (35.8)	0.286
Comorbidities %	9 (30.0)	16 (16.8)	0.116
Diabetes mellitus %	8 (26.7)	8 (8.4)	0.023
TB sputum positive %	4 (13.3)	9 (9.5)	0.51
MDR/XDR %	7 (23.3)	21 (22.1)	0.798
Aspergillus positive %	6 (20.0)	17 (17.9)	>0.999
Duration of surgery (minute)	227.33 ± 97.13	204.11 ± 73.21	0.184
Mean bleeding volume (mL)	843.67 ± 821.26	710.95 ± 864.28	0.346
Type of suture			>0.999
Staple %	29 (96.7)	91 (95.8)	
Suture %	1(3.3)	4(4.2)	

BMI, body mass index; TB, Tuberculosis; MDR/XDR, multidrug resistant tuberculosis/extensively drug-resistant.

**Table 7:** Multivariable logistic regression analysis for key factors associated of postoperative complications in Tuberculosis.

Variables	*P* value	Adjusted OR (95% CI)
Diabetes mellitus	0.013	3.955 (1.335–11.714)

OR, odds ratio; CI, conﬁdence interval.

## Discussion

To date, no expert consensus-based guidelines for the management of destroyed lung have been published. The potential benefits of pneumonectomy for these patients have not been clearly established (12). To address this issue, we investigated differences in clinical characteristics and prognosis of pneumonectomy between TB and non-TB destroyed lung, and identified the risk factors for complications. Our results revealed that the incidence of postoperative complications in the TB group was higher than that in the non-TB group. Importantly, our study suggested that diabetes and duration of surgery were the independent risk factor of postoperative complications after pneumonectomy.

In this study, the incidence of postoperative complications following pneumonectomy was 20.3%, which was consistent with those of previous similar studies (13, 14). Ruan et al. reported a cohort of 113 patients with tuberculosis-destroyed lung who underwent surgical treatment, with a postoperative complication rate of 29.2% (15). This proportion was higher than that observed in our study. Although surgical approaches in Ruan et al.'s study included not only pneumonectomy but also lobectomy, their analysis indicated that the surgical method (lobectomy or pneumonectomy) was not significantly associated with the occurrence of postoperative complications. One plausible explanation for this discrepancy is that the etiology of destroyed lung in their study was limited to tuberculosis. Given that chronic tuberculosis infection impairs patients' physical status, those with tuberculosis-destroyed lung are more prone to postoperative complications compared to patients with destroyed lung of other etiologies (13). Our study, which compared the incidence of postoperative complications between tuberculosis-related and non-tuberculosis-related destroyed lungs, further supports this finding. Therefore, close postoperative monitoring and meticulous perioperative management are necessary for patients with tuberculosis-destroyed lung following pneumonectomy to minimize the incidence of postoperative complications. However, the comparison between the TB and non-TB groups was potentially confounded by differences in baseline characteristics, including diabetes prevalence and surgical complexity. Although we adjusted for these factors in the multivariable analysis, residual confounding cannot be excluded. The higher complication rate in the TB group should be interpreted in the context of these underlying differences rather than being attributed solely to tuberculosis itself.

Bronchopleural fistula is an important and severe complication after pneumonectomy. According to previous studies, its incidence ranges from 3% to 10%. In the present study, the incidence of bronchopleural fistula was 1.9%. The relatively low incidence of bronchopleural fistula (BPF) may be associated with advances in surgical techniques in recent years. In our cohort, most patients underwent bronchial closure using a stapler, while a small proportion of patients with peri-bronchial lymphadenopathy underwent manual bronchial suturing. For cases with unsatisfactory bronchial stump anastomosis, reinforcement with materials such as pleural tissue or intercostal muscle was performed to protect the bronchial stump.

Our study found that diabetes mellitus was significantly associated with an increased risk of postoperative complications in patients undergoing pneumonectomy for destroyed lung. This finding is consistent with the well-established understanding that diabetes impairs immune function and wound healing (16, 17). However, we acknowledge that our data only demonstrate an association rather than proving that glycemic optimization directly reduces complications. Moreover, the small number of diabetic patients (*n* = 16) limit the strength of our conclusions. Nevertheless, our findings highlight the need for vigilant perioperative glucose monitoring and suggest that diabetes should be considered an important risk factor in preoperative risk stratification.

Duration of surgery was identified as an independent risk factor for postoperative complications. However, operative time should be interpreted as a surrogate marker of surgical complexity rather than a direct causal factor. More complex cases—particularly those with dense pleural adhesions, fibrotic hila, and extensive lymphadenopathy—inevitably require longer procedures and are also at higher risk of complications. Therefore, while efforts to optimize surgical efficiency are important, the primary goal should be to perform a thorough and safe dissection, recognizing that prolonged operative time signals increased case complexity and the need for intensified perioperative monitoring.

Postoperative pain associated with thoracotomy may impair effective coughing and deep breathing, potentially contributing to retained secretions and pulmonary infection in the remaining lung. Nevertheless, the muscle-sparing anterolateral thoracotomy used in our center may mitigate this effect to some extent compared with conventional posterolateral thoracotomy.

This study has several limitations. First, the study was retrospectively designed based on a single-center patient cohort. Whether the conclusions of this study can be generalized requires careful consideration. Second, the total sample size, particularly of the non-TB group, was limited, potentially introducing selection and information biases into our conclusions. Third, due to the small number of specific types of postoperative complications, only the risk factors for overall complications could be analyzed, and it was difficult to explore the risk factors for specific complications. Fourth, the limited number of postoperative complications (*n* = 32) restricted the number of variables that could be included in the multivariable regression model to avoid overfitting. Although we adhered to the recommended event-per-variable ratio, the findings should be validated in larger prospective cohorts. Fifth, this study focused primarily on short-term postoperative outcomes and risk factors for complications occurring during hospitalization or within 30 days postoperatively. Long-term outcomes, including symptom relief, recurrence of infection, functional status, quality of life, and long-term survival, were not systematically evaluated. Future prospective studies with extended follow-up are needed to assess the long-term benefits and risks of pneumonectomy for destroyed lung.

In conclusion, our study indicates that pneumonectomy is an effective surgery approach for destroyed lung caused by benign diseases with acceptable risk of complications. Patients with TB destroyed lung have a higher risk of postoperative complications than with non-TB diseases. Diabetes and duration of surgery were the independent risk factor of postoperative complications for the patients with destroyed lung after pneumonectomy. Our findings emphasize the importance of perioperative blood glucose control and shortened surgical duration to prevent adverse outcomes for these patients.

## Data Availability

The original contributions presented in the study are included in the article/Supplementary Material, further inquiries can be directed to the corresponding author/s.
